# Efficacy of high-dose vitamin D3 supplementation in vitamin D deficient pregnant women with multiple sclerosis: Preliminary findings of a randomized-controlled trial

**Published:** 2015-04-04

**Authors:** Masoud Etemadifar, Mohsen Janghorbani

**Affiliations:** 1 Department of Neurology, School of Medicine, Isfahan University of Medical Sciences, Isfahan, Iran; 2 Department of Epidemiology and Biostatistics, School of Medicine, Isfahan University of Medical Sciences, Isfahan, Iran

**Keywords:** Vitamin D, Multiple Sclerosis, Pregnancy, Iran

## Abstract

**Background: **The aim of this preliminary study was to assess the safety and efficacy of high-dose oral vitamin D3 supplementation during pregnancy in women with multiple sclerosis (MS) in Isfahan, Iran.

**Methods:** In a single center open-label randomized, controlled clinical Phase I/II pilot study, 15 pregnant women with confirmed MS with low serum 25-hydroxyvitamin D (25(OH)D) levels were randomly allocated to receive either 50,000 IU/week vitamin D3 or routine care from 12 to 16 weeks of gestation till delivery. The main outcome measures were mean change in serum 25(OH)D levels, expanded disability status scale (EDSS) score, and number of relapse events during pregnancy and within 6 months after delivery.

**Results: **Average serum 25(OH)D level at the end of trial in vitamin D3 supplemented group was higher than routine care group (33.7 ng/mL vs. 14.6 ng/ml, P < 0.050). In vitamin D3 group, the mean EDSS did not changed 6 months after delivery (P > 0.050)**, **whereas in routine care group, the mean EDSS increased from 1.3 (0.4) to 1.7 (0.6) (P < 0.070). Women in vitamin D3 group appeared to have fewer relapse events during pregnancy and within 6 months after delivery. No significant adverse events occurred.

**Conclusion: **Adding high dose vitamin D3 supplementation during pregnancy to routine care of women with MS had significant effect on the serum 25(OH)D levels, EDSS and number of relapse events during pregnancy and within 6 months after delivery.

## Introduction

Multiple sclerosis (MS), a demyelinating disease of unknown cause, is the most common in women of childbearing age, and these women are most affected by low vitamin D levels.^1^^,^^2^ The increased physiological needs in pregnancy and more indoor activity are also important risk factors increasing the vulnerability to vitamin D deficiency in these women. Low serum level of 25-hydroxyvitamin D (25(OH)D) which is the biologically inactive storage form of vitamin D3 appears to be a risk factor for both MS development and the MS course.^3^^-^^6^ Some studies provide a direct correlation between vitamin D3 intake and serum 25(OH)D levels in non-pregnant MS patients.^5^^,^^7^^-^^9^ No well-designed clinical trial is available adding high dose vitamin D3 supplementation to routine care in pregnant women with MS.

Vitamin D3 plays an important role in bone formation and mineral homeostasis. Some in-vitro and animal studies have also suggested that vitamin D3 has anti-inflammatory actions, including enhanced Th2 and decreased Th1 cytokines production, dendritic cell effects and enhanced macrophage phagocytosis.^10^^-^^12^ There is accumulating evidence for a possible protective role of vitamin D3 in the development and disease course of MS.^7^^,^^13^^-^^16^ Several studies have reported that low serum 25(OH)D levels may increase the risk of relapses in non-pregnant patients with MS.^3^^,^^8^^,^^17^ Whether high dose vitamin D3 is also effective in treating pregnant women with MS and low serum 25(OH)D level is not known. We hypothesize that high dose vitamin D3 supplementation during pregnancy are safe, increases the serum 25(OH)D level, changed expanded disability status scale (EDSS) and number of relapse events in pregnant women with MS.

## Materials and Methods

This was a single center open-label exploratory Phase I/II randomized parallel-group clinical trial to evaluate the effect of oral high-dose vitamin D3 on the serum 25(OH)D levels, EDSS, and number of relapse events in pregnant women with MS.

The original study sample comprised of 52 consecutive clinically definite MS patients who intended to be pregnant and sought treatment at our MS outpatient clinics of Isfahan University of Medical Sciences, Iran, between July 2011 and December 2012: of these 15 became pregnant and returned for follow-up. Entry criteria were women age between 20 and 40 years with a magnetic resonance imaging, clinical or laboratory-supported diagnosis of definite MS,^18^ stable neurological functioning for at least 1-month prior to study entry, and an EDSS^19^ score ≤ 6, serum 25(OH)D level < 20 ng/ml^20^ and a willingness to continue current medications for the duration of the study. Assessments of serum 25(OH)D levels was carried out routinely as part of the clinical management of MS and used to detect vitamin D3 insufficiency. Serum 25(OH)D was measured using a commercially available radioimmunoassay kit (DiaSorin, Stillwater, MN, USA). Exclusion criteria were evidence of substantial abnormalities in psychiatric, cardiac, endocrinological, hematologic, hepatic, renal or metabolic functions, vitamin D3 supplement, and any condition predisposing to hypercalcemia, nephrolithiasis, and renal insufficiency as determined by history, physical examination, and screening blood tests. Patients who demonstrated poor compliance with instructions to take vitamin D3, or who failed to attend for follow-up visits, abortion, not become pregnant and 25(OH)D measurements during the study were also excluded. Tenets of the current version of the Declaration of Helsinki and the guidelines of the International Conference on Harmonization of Good Clinical Practice were followed, the study protocol was approved by the Ethics Committee of Isfahan University of Medical Sciences and the nature of the trial was explained to all participants. After a detailed discussion with the neurologist, patients made a final decision, and each participant provided written informed consent.

**Figure 1:**
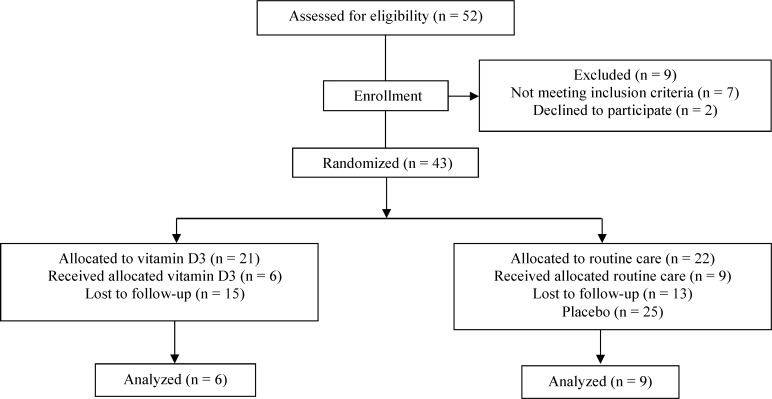
Design of the trial to compare oral vitamin D3 (50,000 IU/week) versus routine care in pregnant patients with multiple sclerosis

A total of 52 consecutive patients were eligible for the study. Thirty-seven patients were excluded because, they refused entry, or they did not meet the inclusion criteria, or failed to become pregnant, or did not attend for a follow-up visit. Fifteen pregnant patients completed the study without interruption. Patients were randomized according to a preexisting list produced by a computer program. The first treatment group received a single dose of 50,000 IU vitamin D3 (trade name Vitamin D3**,** Zahravi Pharm. Co. Tabriz, Iran) per week in the form of oral pearls from 12 to 16 weeks of gestation and continued during pregnancy . The second group received routine care. Compliance with the study treatment was verified by asking the patients about missed doses and by counting unused pearls. All patients underwent pretreatment evaluation to record demographic data, complete neurologic and medical history, the finding of physical and neurologic examination, and previous treatment. Figure 1 illustrates the patient allocation algorithm. In the final sample of participants, mean (standard deviation) age was 29.1 (3.5) years (range 22 to 36 years).

The trial was open-label in that both patients and investigators were aware of the type of treatment each patient received. Participants were evaluated by a qualified neurologist (ME) at baseline and every 8 weeks after the start of the therapy till delivery and 6 months after delivery to evaluate the development of side effects of the medications, compliance, and disease activity. The number of relapses, the proportion of patients free from relapses, the EDSS, and other medical events were recorded at baseline and each visit. Acute relapse was defined as the appearance of a new neurological symptom or severe deterioration in a pre-existing symptom that lasted for at least 24 h in the absence of fever/infection and caused an increase of at least 1 point in EDSS^21^ and confirmed by the treating physician. Patients experiencing relapses received steroids (IV methylprednisolone or oral prednisone) as deemed appropriate by treating physician.

The primary outcome measure was mean changes in serum level of 25(OH)D from baseline to 6 months after delivery. Mean changes in the EDSS and number of relapses were also measured for both groups. Safety and tolerability were assessed by vital signs, safety lab, ECG, and adverse reporting.

Since available data on the beneficial therapeutic effects of high dose vitamin D3 supplementation on pregnant women with MS are not sufficient for an exact statistical sample size calculation the study was designed as pilot study with a priori determined sample size of 7 patients per intervention arm.

Between-group comparison of changes was made using Mann–Whitney U-tests. Within group comparisons were undertaken using Wilcoxon sign-rank test, to determine differences between baseline and 6 months after delivery assessment of serum level of 25(OH)D, EDSS, and relapse events. Comparisons between proportions were undertaken using Fisher’s exact test. Results are expressed as mean [standard deviation (SD)] and P < 0.050 was considered statistically significant. All statistical tests were two-sided. The analyses were undertaken using SPSS for Windows (version 18.0, SPSS Inc., Chicago, IL, USA).

## Results

Fifteen patients who met the entry criteria were enrolled in the study. All 15 patients who completed treatment were available for follow-up at 6 months after delivery. The two treatment groups were generally well-matched at baseline with regard to age, age at pregnancy, onset of MS to pregnancy, EDSS before pregnancy, number of relapses/year prior to the trial and other characteristics. With respect to serum 25(OH)D level, women in the vitamin D3 supplemented group had slightly but significantly lower serum 25(OH)D level (P < 0.050). Mean ± SD serum 25(OH)D level at the start of treatment was 15.3 ± 2.9 ng/ml in the vitamin D3 group and 18.3 ± 1.9 ng/ml in the routine care group. Mean ± SD age in the vitamin D3 and routine care groups were 27.7 ± 2.4 and 30.0 ± 3.9 years, respectively. Five women in routine care group relapsed within 6 months after delivery and 4 women relapsed during pregnancy. In vitamin D3 group, no women relapsed within 6 months after delivery (Table 1).

High dose vitamin D3 supplementation was tolerated well, and no adverse events with the use of vitamin D3 were reported. There were no instances of urinary dysfunction or a symptomatic nephrolithiasis. No disturbances of cardiac rhythm were seen. In both arms of the study, patients received similar therapies before/during trial.

Changes in mean serum 25(OH)D level, EDSS, and relapse events before and after receiving vitamin D3 supplementation or routine care are shown in table 2. As expected, in vitamin D3 group, the average serum 25(OH) level increased significantly. Of the six patients treated with vitamin D3, the mean ± SD serum 25(OH)D level increased from 15.3 ± 2.9 ng/ml at baseline to 33.7 ± 15.2 ng/ml at the end of study period (P < 0.050). Whereas in routine care group, the average serum 25(OH)D level decreased significantly. In the nine patients treated with routine care, the mean ± SD serum 25(OH)D level decreased from 18.3 ± 1.9 ng/ml at baseline to 14.6 ± 1.3 at the end of study period (P < 0.001). Six months after delivery, average increase in serum 25(OH)D level between vitamin D3 and routine care groups was 19.1 [95% confidence interval (CI), 8.3, 29.9 ng/ml (around 57%)], indicating there is evidence of an effect on the serum 25(OH)D level in patients who received high-dose vitamin D3 supplementation compared to those who received the routine care.

In vitamin D3 group, the mean EDSS did not change (P > 0.050). Whereas in routine care group, the mean EDSS increased from 1.3 ± 0.4 to 1.7 ± 0.6 (P < 0.070). There was a significant difference in the EDSS at the end of the study period between the vitamin D3 and routine care groups (mean difference, −0.6; 95% CI −1.2, −0.1). The mean EDSS was lower in vitamin D3 supplemented group than in the routine care group (1.1 vs. 1.7, P < 0.050) (Table 2).

In both vitamin D3 and routine care groups, the mean number of relapses within 6 months after delivery significantly decreased. Mean number of relapses in vitamin D3 group decreased from 1.3 to 0.0, while in routine care group, it also decreased, from 1.1 to 0.4. There was a significant difference in the average number of relapses within 6 months after delivery between the vitamin D3 and routine care groups (mean difference, −0.4; (95% CI −0.9, 0.2; P < 0.060) (Table 2). In routine care group, relapses during pregnancy were observed in five cases and relapses after delivery were observed in four cases. 

**Table 1:** Characteristics of pregnant women with multiple sclerosis who received high-dose vitamin D3 or routine care at baseline.

**Characteristics**	**Treatment group at baseline**				**P** *
	**Vitamin D3 (n = 6) **		**Routine care (n = 9) **		
	**Mean ± SD**	**n (%)**	**Mean ± SD**	**n (%)**	
Age (year)	27.7 ± 2.4		30.0 ± 3.9		NS
Age at pregnancy (year)	25.3 ± 2.4		27.9 ± 3.9		NS
Onset of MS to pregnancy (year)	3.3 ± 2.2		5.0 ± 3.9		NS
Relapse rate/year before pregnancy	1.3 ± 0.5		1.1 ± 0.4		NS
EDSS before pregnancy	1.2 ± 0.3		1.3 ± 0.4		NS
Serum 25(OH)D level (ng/ml)	15.3 ± 2.9		18.3 ±1.9		< 0.050
Relapse during pregnancy		0.0 (0.0)		5.0 (55.6)	< 0.050
6-7 months		-		2.0 (22.2)	NS
7-8 months		-		3.0 (33.3)	NS
Relapse up to 6 months after delivery		0.0 (0.0)		4.0 (44.4)	NS
0-1 month		-		1.0 (11.1)	NS
2-3 months		-		1.0 (11.1)	NS
3-4 months		-		2.0 (22.2)	NS

* Differences in mean (Mann–Whitney U-test) or percentage (Fisher exact test) values of variables between vitamin D3 and routine care; NS: Non-significance; MS: Multiple sclerosis; EDSS: Expanded Disability Status Scale; 25(OH)D: 25-hydroxyvitamin D; SD: Standard deviation

**Table 2:** Comparison of serum 25(OH)D level, expanded disability status scale (EDSS), and relapses in 15 pregnant women with multiple sclerosis before and 6 months after delivery with vitamin D3 supplementation and routine care

**Treatment group**	**N**	**Baseline (mean ± SD)**	**6 months after delivery (mean ± SD)**	**P** *
Serum 25(OH)D level				
Vitamin D3	6	15.3 ± 2.9	33.7 ± 15.2	< 0.050
Routine care	9	18.3 ±1.9	14.6 ± 1.3	< 0.001
P**	-	< 0.05	< 0.01	-
EDSS				
Vitamin D3	6	1.2 ±0.3	1.1 ± 0.2	NS
Routine care	9	1.3 ± 0.4	1.7 ± 0.6	NS
P**	-	NS	< 0.05	-
Relapses				
Vitamin D3	6	1.3 ± 0.5	0.0 ± 0.0	< 0.010
Routine care	9	1.1 ± 0.4	0.4 ± 0.5	< 0.010
P**	-	NS	NS	-

* Differences in mean values of variables between baseline and 6 months after delivery (Wilcoxon sign-rank test);

** Differences in mean values of variables between vitamin D3 and routine care (Mann–Whitney U-test); EDSS: Expanded disability status scale; NS: Non-significance; 25(OH)D: 25-hydroxyvitamin D; SD: Standard deviation

## Discussion

In this exploratory study we found that adding high dose vitamin D3 supplementation during pregnancy to routine care of women with MS had significant effect on the serum 25(OH)D levels, EDSS, and number of relapse events during pregnancy and within 6 months after delivery. The serum 25(OH)D levels before supplementation were about 17 ng/ml in our pregnant women with MS. Therefore, the primary aim of our study was to bring the serum 25(OH)D levels of the women in vitamin D3 supplemented group to > 40 ng/ml zone, which is often considered the critical physiological lower limit for protect patients with MS.^9^^,^^22^^-^^24^ After high-dose vitamin D3 supplementation, an average serum level of 33.7 ng/ml reached. While serum level of routine care group decreased. No unusual or unexpected safety risks were found with high-dose vitamin D3 supplementation in our pregnant women with MS 6 months after delivery. Previous studies have shown that high dose vitamin D3 is fairly safe in non-pregnant MS patients.^13^^,^^25^

We paid particular attention to the EDSS and number of relapses since the anti-inflammatory, and immunomodulatory effects of vitamin D3 could particularly influence these variables. Our findings showed a significant decreased EDSS and mean number of relapses during pregnancy in vitamin D3 supplemented group.

While the efficacy of vitamin D3 supplementation for treatment of MS in non-pregnant adults has been examined in a few small non-controlled trials with variable results,^14^^-^^16^^,^^26^ only one open-label randomized controlled trial conducted over 52 weeks, which treated 25 patients with escalating doses of vitamin D compared with control.^13^ This trail provided some evidence of the potential benefit of the high dose vitamin D3 on several outcomes i.e. the annualized relapse rate, EDSS score, suppression of T-cell proliferation and illustrated a measure of comparative safety in the relative absence of any adverse events or of high serum calcium level over the study period. Our findings in pregnant women with MS are consistent with this study in non-pregnant MS adult patients that found high-dose vitamin D3 (~10,000 IU/day) is safe, with evidence of immunomodulatory effects.^13^ To the best of our knowledge, no study is available adding high dose vitamin D3 to routine care in pregnant women with MS.

The mechanisms whereby vitamin D3 supplementation exerts positive effects on MS course in pregnant women are not clear because there is not enough research in this area. Similar to non-pregnant MS patients, anti-inflammatory and immunomodulatory effects are probably most important. However, low serum 25(OH)D appears to be an important modifiable external risk factor for MS course in pregnant women with MS. Relative low serum 25(OH)D levels during pregnancy may worsen the course of MS by influencing metabolic pathways in the myelinating central nervous system that we do not understand at present. Furthermore, prevention of demyelination has also been demonstrated in a model of toxic demyelination.^27^

Albeit, this study is only controlled trials to date of effect of high-dose oral vitamin D3 on the serum 25(OH)D level, EDSS, and number of relapses in pregnant women with MS the sample sizes was small, and was limited by the loss to follow-up of 37/52 of the original baseline cohort. Hence, selection and volunteer bias cannot be ruled out. The efficacy should, therefore, be tested in a larger sample. The present results clearly need to be replicated and extended across multiple centers and investigators.

Serum levels of 25(OH)D are often quite low in MS patients.^5^ Thus, we expect that low serum levels of 25(OH)D will be detected in pregnant women with MS. From an ethical point of view and bearing in mind the importance of vitamin D3 for bone metabolism, anti-inflammatory, and immunomodulatory effect, it would be difficult not to supplement these women with vitamin D3. On the other hand, we do not know, at which doses or serum levels vitamin D3 start to have anti-inflammatory and immunomodulatory effects. Thus, we choose the maximum dose for which sufficient safety data are available, which currently corresponds to 10,000 IU/day.^28^ Thus, it appears wise to supplement all pregnant women with MS currently in a state of vitamin D3 deficiency or insufficiency in order to bring their serum 25(OH)D to >40 ng/ml level which might be neurologically beneficial for the course of the disease.

Although vitamin D deficiency or insufficiency is thought to be common among pregnant women, and substantial evidence supports the safety of even large dose of vitamin D3 in non-pregnant individuals, such evidence is based on studies of limited size and duration and there is no evidence of its usefulness and safety in pregnant women. A recent Cochrane review on vitamin D supplementation for women during pregnancy conclude that the clinical significance of vitamin D supplementation in pregnancy and the potential use of this intervention as a part of routine antenatal care are yet to be determined as the number of high-quality trials and outcomes reported is too limited to draw conclusions on its usefulness and safety. There is no evidence that vitamin D supplementation prevents pre-eclampsia, gestational diabetes, impaired glucose tolerance, caesarean section, gestational hypertension, or death in the mothers; or preterm birth, stillbirth, neonatal death, neonatal admission to intensive care unit, newborns with low Apgar score or neonatal infection. The number of trials and outcomes reported are too limited, and, in general, are of low quality, to draw conclusions on the usefulness and safety of this intervention as a part of routine antenatal care. Further rigorous randomized trials are required to evaluate the role of vitamin D supplementation in pregnancy.^29^ In addition, it is well-established that pregnant women with MS have a low risk of relapse and that lactation does not increase the risk of relapses. There is also no evidence that hormonal effects of pregnancy or lactation are different in women with MS compared with healthy women.^30^^,^^31^

The best level of 25(OH)D for health is uncertain.^22^ Many experts believe that blood levels of 25(OH)D > 40 ng/ml are adequate.^9^^,^^22^^-^^24^ Some investigators also suggested that levels higher than 40 ng/ml may further help protect patients with MS.^9^^,^^17^^,^^23^^,^^24^^,^^32^^,^^33^ The US Institute of Medicine has determined that concentrations greater than 50 nmol/l or 20 ng/ml are adequate based on the current studies available.^34^ However, there is controversy regarding the 25(OH)D levels that are considered adequate or optimal for overall health. It has been suggested that a supplemental dose of vitamin D of 1000 to 1600 IU (25-40 µg/d) might be necessary to achieve the optimal level of this vitamin in the body.^33^ This dose is expected to raise serum 25(OH)D by 1.2 nmol/l for every µg (40 IU) of vitamin D_3_ given orally to individuals with low 25(OH)D levels; those with higher baseline concentrations would have smaller increments with the same dose.^33^ However, the dose of vitamin D needed to have an effect during pregnancy or to prevent or treat vitamin D deficiency is not clear. Some researchers have suggested that doses around 1000 IU/d may be needed in order for pregnant women to maintain a blood concentration of vitamin D of more than 50 nmol/l (20 ng/ml).^35^ Others have suggested providing vitamin D as weekly doses of 5000 IU (125 µg/week)^36^ or a single dose of 200,000 IU (5 mg) or greater (9).^37^

Weekly high-doses of up to 280,000 IU vitamin D3 and long-term treatment with a mean weekly dose of at least 70,000 IU for 36 weeks have been well-tolerated in non-pregnant adults.^6^^,^^13^ Furthermore, non-pregnant patients with MS tolerated a pilot dose-escalation trial up to 40,000 IU/day.^13^ A daily supplement of 10,000 IU of vitamin D3 is considered advisable for all adults with normal renal function^22^^,^^28^ and this dose should be routinely recommended to all women, particularly women with insufficient serum 25(OH)D levels, during pregnancy and lactation. Therefore, our study suggests that the dose of 50,000 IU/week vitamin D3 in patients with insufficient serum 25(OH)D levels, which is well above current recommendation for pregnant and lactating women, may be considered relatively safe. Although this trial was not powered nor blinded to properly address clinical outcomes, we observed that clinical outcomes appeared to favor the treatment group.

## Conclusion

This exploratory Phase I/II comparative trial of vitamin D3 supplementation and routine care showed that adding vitamin D3 to routine therapy had significant effect on the serum 25(OH)D levels, EDSS and number of relapse events within 6 months after delivery. Further studies with larger sample and longer follow-up are needed to be able to recommend routine high-dose vitamin D3 supplementation in pregnant women with MS.

## Notes:

**How to cite this article: **Etemadifar M, Janghorbani M. Efficacy of high-dose vitamin D3 supplementation in vitamin D deficient pregnant women with multiple sclerosis: Preliminary findings of a randomized-controlled trial. Iran J Neurol 2015; 14(2): 67-73.
